# Anterior Papillary Muscle Rupture due to Acute Myocardial Microinfarction of the Small High Lateral Branch

**DOI:** 10.1155/2022/7149724

**Published:** 2022-07-08

**Authors:** Takanori Kono, Kazuyoshi Takagi, Shinya Negoto, Eiki Tayama

**Affiliations:** Department of Surgery, Division of Cardiovascular Surgery, Kurume University School of Medicine, 67 Asahi-machi, Kurume City, Fukuoka, Japan

## Abstract

Papillary muscle rupture is a catastrophic condition, of which most cases manifest posterior papillary muscle rupture. Anterior papillary muscle rupture is a rare condition. Particularly, anterior papillary muscle rupture due to acute myocardial microinfarction of the small high lateral branch is extremely rare. Since papillary muscle rupture can occur even in such a small branch or small area due to myocardial infarction, echocardiographic and/or ventriculographic confirmation is essential in cases of sudden worsening of heart failure. Herein, we report the case of anterior papillary muscle rupture with a good outcome. A 61-year-old man was admitted to our hospital with cardiogenic shock. Echocardiography revealed severe mitral regurgitation due to papillary muscle rupture. Coronary angiography demonstrated high-grade stenosis in the high lateral branch. However, no other significant stenoses were found. Emergency mitral valve replacement was performed. The patient was discharged 19 days after surgery without any complications.

## 1. Introduction

The incidence of papillary muscle rupture (PMR) in cases of acute myocardial infarction (AMI) is approximately 1%–5% [1]. Most of these cases are posterior PMR, and anterior PMR is a very rare condition [2, 3]. Cardiogenic shock in patients with PMR occurred in about 85% of hospitalized cases of AMI, often requiring mechanical circulatory support (MCS) devices [4]. Surgical outcomes of PMR remain poor with a reported early mortality rate of 25–40% [1, 5]. Herein, we report the case of anterior PMR with good outcomes. This case was diagnosed using transthoracic and transesophageal echocardiography and managed with preoperative extracorporeal membrane oxygenation (ECMO), Impella (Abiomed, Danvers, MA, USA), and prompt mitral valve replacement (MVR).

## 2. Case Presentation

**Figure 1:**
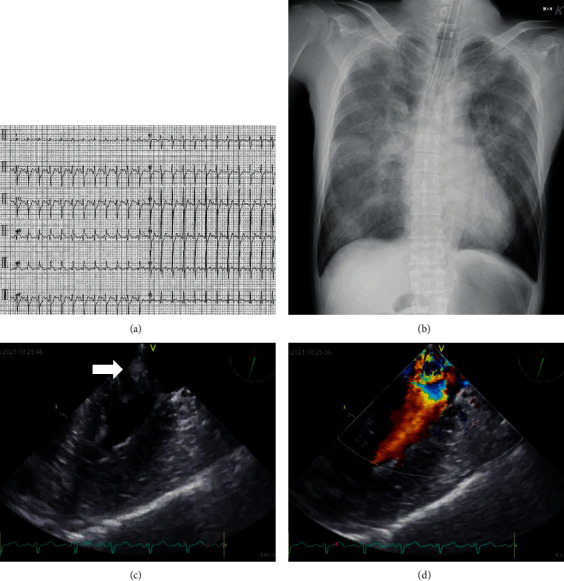
Preoperative electrocardiogram reveals ST-segment elevation in leads I, aVL, and aVR (a). Preoperative chest radiographs reveal severe pulmonary congestion (b). Preoperative transesophageal echocardiography reveals severe mitral regurgitation due to papillary muscle rupture (c, d). The white arrow indicates the ruptured anterior papillary muscle.

**Figure 2:**
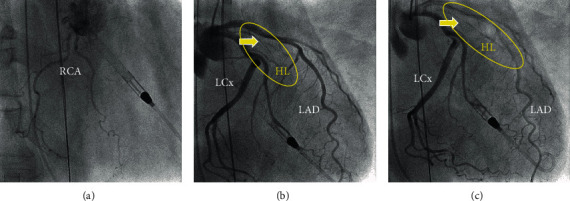
Coronary angiography reveals no significant stenosis in the right coronary artery (a) and left anterior descending artery and circumflex arteries (b), and stenosis of the high lateral branch (c) is observed. The yellow circle indicates the high lateral branch, and the yellow arrow indicates stenosis. LAD: left anterior descending artery; LCx: left circumflex artery; RCA: right coronary artery.

**Figure 3:**
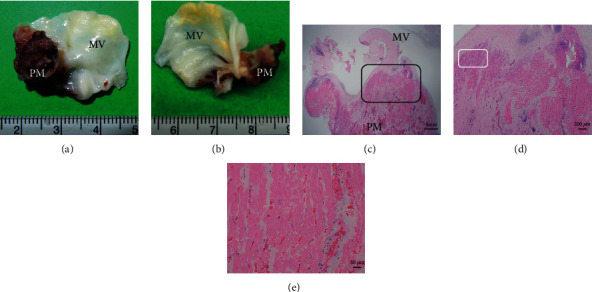
Resected specimen reveals complete rupture of the anterior papillary muscle (a, b). The pathological findings of the resected anterior papillary muscle reveal ischemic changes and necrosis of the myocytes (c–e). The black quadrangle indicates the area of (d), and the white quadrangle indicates the area of (e). MV: mitral valve; PM: papillary muscle.

A 61-year-old man was transferred to our hospital with complaints of fever and chills. His condition progressed to a state of shock during examination and was transported to our emergency center due to cardiogenic shock and septic shock. The patient had low systolic blood pressure, 40 mmHg; fast heart rate, 143 beats/min; and high temperature, 40.6°C. A late systolic murmur was heard at the cardiac apex. Arterial blood gas analysis during oxygen ventilation at 10 L/min revealed the following: pH, 6.952; PaO_2_, 59.8 mmHg; PaCO_2_, 80.9 mmHg; HCO^3-^, 17.0 mEq/L; and lactic acid, 12.4 mmol/L. ECMO was immediately established. Electrocardiogram revealed ST-segment elevation in leads I, aVL, and aVR (Figure 1(a)). Chest radiographs showed severe bilateral pulmonary congestion (Figure 1(b)). Laboratory tests of the blood revealed the following: white blood cell count, 20,200/*μ*L; C-reactive protein, 4.27 mg/dL; creatine phosphokinase/creatine phosphokinase-MB isozyme, 687/54 IU/L; aspartate transaminase/alanine transaminase, 121/36 IU/L; lactate dehydrogenase, 616 IU/L; and N-terminal (NT) prohormone B-type natriuretic peptide, 5249.3 ng/L. Results of nasopharyngeal swab tests for coronavirus disease 2019 (COVID-19) and flu were negative. Transthoracic and transesophageal echocardiography demonstrated severe mitral regurgitation due to PMR (Figures 1(c) and 1(d), Videos 1 and 2). Coronary angiography was performed after Impella CP was placed via the left femoral artery for further hemodynamic support and left ventricle unloading (ECMO flow, 2.5 L/min; Impella performance level, P6). Activated clotting time was maintained around 200 seconds. A high-grade stenosis (99%) was noted in the high lateral branch (HL). However, no other lesions with significant stenosis were noted (Figure 2, Video 3). Emergency surgery was performed. Although lateral wall infarction was suspected, gross ischemic changes were not observed intraoperatively. The anterior leaflet of the mitral valve (A1-2) and the posterior leaflet (P1) with the ruptured anterior papillary muscle (PM) had prolapsed into the left atrium. MVR was performed using a 27 mm mechanical valve (St. Jude Medical, St Paul, MN, USA). Pathological examination of the resected PM revealed ischemic changes and necrosis of the myocytes almost entirely with features such as wavy fibers, loss of nuclei, and striations (Figure 3). The patient was weaned off ECMO and the Impella after MVR and returned to the intensive care unit on intra-aortic balloon pumping (IABP) support. IABP was removed on postoperative day (POD) 3, and the patient was discharged on POD19 without any complications.

## 3. Discussion

PMR is a rare but catastrophic complication. Its reported incidence is 1%–5% of all cases of AMI with an onset of 2–7 days after AMI [1, 6]. More than 80% of patients with PMR develop cardiogenic shock and die within 24 hours [7]. The consequent estimated mortality has been reported to be approximately 80%–90% with medical treatment and 19%–30% with emergency surgery [2, 8]. The poor preoperative conditions in such patients may contribute to poor surgical outcomes. Therefore, early diagnosis and adequate preoperative management are important.

In addition to PMR, other potentially fatal complications of myocardial infarction (MI) include left ventricular rupture and ventricular septum perforation. In this case, we were able to diagnose the patient with PMR using transthoracic and transesophageal echocardiography. Otherwise, the sensitivity of echocardiography for diagnosing complications after AMI showed marked variation [9]. Some reports indicated that left ventriculography (LVG) was useful for identifying mechanical complications after AMI [10]. Performing LVG along with coronary angiography can provide immediate information of complications in the catheter laboratory and is important for preoperative patient assessment to consider additional surgical procedures. We better might have considered performing LVG to rule out other complications in this case.

Since overall incidence of cardiogenic shock in patients with PMR is extremely high, appropriate MCS device use is needed. Although IABP is easy to insert and reported to be used in more than 90% of PMR cases [4], the effect of single use of IABP is not sufficient in these severely ill patients. Despite ECMO being a good treatment option, it might worsen mitral regurgitation due to increased afterload [4]. Impella is a reasonable option for left ventricular unloading and reduction in mitral regurgitation. However, using Impella alone cannot improve hypoxia due to pulmonary edema. The usefulness of ECMO and Impella in preoperative management has been reported [7], and the prompt introduction of Impella in addition to ECMO, in this case, is thought to have contributed to the good outcome. Although there have been reports of good results of combined ECMO and Impella following elective surgery, we suggest that prompt introduction of MCS soon after diagnosis and emergency surgery may improve the outcomes. Furthermore, MCS is useful for left ventricular unloading in the perioperative period after PMR repair. However, due to the high risk of bleeding with Impella, IABP is better for postoperative management if cardiac function is adequate.

PMR is 6–12 times more common in the posterior papillary muscle (PM) compared with the anterior PM because the posterior PM typically has a single blood supply from the right coronary artery or left circumflex artery (LCx). The anterior PM has dual blood supply from the left anterior descending artery and LCx [2, 3]. However, on rare occasions, the anterior PM may have a single blood supply, which can result in anterior PMR. Furthermore, PMs are located on the most endocardial part of the heart. Therefore, PMR can occur even in subendocardial MI and small-sized MI [2, 3]. Nishimura et al. reported that PMR was more likely to occur in smaller infarctions because a relatively good ventricular function may result in a greater shearing force at the site of potential rupture [3]. In the present patient, MI of the HL was not visible macroscopically because the patient had a microinfarction on the small HL branch. However, pathological findings revealed PMR due to MI. To the best of our knowledge, anterior PMR is rare, and anterior PMR due to acute myocardial microinfarction of the small HL is extremely rare. Kuwata et al. reviewed previous reports of 15 cases of anterior PMR in Japan. They reported that the responsible lesions were in the LCx in 10 cases, high lateral branch in 3 cases (two-vessel disease in 1 case), and diagonal branch in 2 cases [11]. Since PMR can occur even in such a small branch or small area of MI, echocardiographic confirmation is essential in cases of sudden worsening of heart failure.

We chose MVR as our surgical strategy. Mitral valve repair has been reported to yield good results in selected cases with an operative mortality rate of 0%, 15 years freedom from moderate or severe mitral regurgitation [12]. On the other hand, repair of necrotic PMs is often difficult. In addition, there is a concern that the extent of necrosis may lead to recurrence of MR. The fact that valve replacement is actually chosen in many reported PMR cases may indicate that MVR is a reasonable option, given that the patient's goal for improving hemodynamics by eliminating mitral regurgitation [13].

In conclusion, we observed good outcomes in our patient with anterior PMR due to AMI of the HL due to rapid diagnosis, immediate introduction of MCS, and appropriate timing of surgery.

## Supplementary Materials

Supplementary Materials Videos 1 and 2: preoperative transthoracic (Video 1) and transesophageal (Video 2) echocardiography reveals severe mitral regurgitation due to papillary muscle rupture. Video 3: coronary angiography reveals a high-grade stenosis of the high lateral branch. However, no other lesions with significant stenosis were noted.
